# Nanosonosensitizers With Ultrasound-Induced Reactive Oxygen Species Generation for Cancer Sonodynamic Immunotherapy

**DOI:** 10.3389/fbioe.2021.761218

**Published:** 2021-09-30

**Authors:** Danling Cheng, Xiaoying Wang, Xiaojun Zhou, Jingchao Li

**Affiliations:** ^1^ Shanghai Engineering Research Center of Nano-Biomaterials and Regenerative Medicine, College of Chemistry, Chemical Engineering and Biotechnology, Donghua University, Shanghai, China; ^2^ Xuhui District Center for Disease Control and Prevention, Shanghai, China

**Keywords:** reactive oxygen species, nanosonosensitizers, ultrasound, immunotherapy, sonodynamic therapy

## Abstract

Immunotherapy is a promising therapeutic strategy for cancer, while it has been demonstrated to encounter the issues of low immune responses and underlying immune-related adverse events. The sonodynamic therapy (SDT) that utilizes sonosensitizers to produce reactive oxygen species (ROS) triggered by ultrasound (US) stimulation can be used to ablate tumors, which also leads to the induction of immunogenic cell death (ICD), thus achieving SDT-induced immunotherapy. Further combination of SDT with immunotherapy is able to afford enhanced antitumor immunity for tumor regression. In this mini review, we summarize the recent development of nanosonosensitizers with US-induced ROS generation for cancer SDT immunotherapy. The uses of nanosonosensitizers to achieve SDT-induced immunotherapy, combinational therapy of SDT with immunotherapy, and combinational therapy of SDT with multiple immunotherapies are briefly introduced. Furthermore, the current concerns and perspectives for the development and further clinical applications of these nanosonosensitizers for SDT-combined immunotherapy of cancer are discussed.

## Introduction

Immunotherapy including cancer vaccines, immune checkpoint blockade therapy, and adoptive T-cell therapy has emerged as a promising strategy for cancer treatment (Pardoll, 2012; Kuai et al., 2017; Fraietta et al., 2018). Different from some traditional therapeutic modalities that directly target tumor cells, immunotherapy achieves the treatment of tumors by boosting the body’s systemic immune responses and triggering the activation of immune cells (Del Paggio, 2018). In such a unique way, immunotherapy can not only eradicate localized tumors but also remove distant metastatic tumors and even prevent tumor recurrence by forming long-term immune memory (Nam et al., 2019; Li et al., 2021a; Vitale et al., 2021). Currently, immunotherapy has shown ideal antitumor efficacy in a subset of clinical patients, which benefits from the approval of various immunotherapeutic agents by the U.S. Food and Drug Administration (FDA) (Zhang and Pu, 2020). However, only a few patients are currently proven to be responsive to immunotherapy, and a substantial fraction of them receiving treatments will develop immune-related adverse events (irAEs) (Boutros et al., 2016; Chiang et al., 2018; Song et al., 2018; Eun et al., 2019).

It has been extensively demonstrated that other treatment methods (such as chemotherapy, photodynamic therapy, photothermal therapy, and radiotherapy) can also trigger immunity by inducing immunogenic cell death (ICD) with the release of tumor-associated antigens (TAAs) (Ma et al., 2019b; Chen et al., 2019; Jin et al., 2021; Zhao et al., 2021). Moreover, these treatments can convert non-immunogenic “cold” tumor microenvironment to a “hot” one, which contributes to enhanced therapeutic efficacy of immune checkpoint blockade (Irvine and Dane, 2020). Nevertheless, obvious systemic side effects of chemotherapy/radiotherapy and shallow tissue penetration of photodynamic/photothermal therapy limit their efficacy for tumor treatment (Maruoka et al., 2018; Ma et al., 2019c). In contrast, the sonodynamic therapy (SDT) utilizing sonosensitizers to generate reactive oxygen species (ROS) under ultrasound (US) irradiation for tumor ablation shows negligible toxicity to normal tissues and can overcome the tissue penetration limitation due to its tissue penetration depth of >10 cm (Zhang et al., 2016; Li et al., 2018; Ma A. et al., 2019). Therefore, the combination of SDT with immunotherapy has been considered as a promising strategy for cancer treatment. In this regard, a class of nanosonosensitizers (such as small molecular sonosensitizer-based nanoparticles and inorganic nanomaterials) with ROS generation ability have been developed for this purpose.

Herein, we summarize the recent development of nanosonosensitizers with US-induced ROS generation for their application in cancer treatment by combining SDT with immunotherapy. In the following, the key roles of nanosonosensitizer-mediated SDT in inducing ICD and thus promoting antitumor immunity are discussed. The combinations of SDT with different types of immunotherapy for tumor remission are then introduced. A brief discussion of existing concerns and perspectives for the development of nanosonosensitizers toward clinical translation of SDT immunotherapy are also given.

### Sonodynamic Therapy–Triggered Antitumor Immunity

In addition to direct ablation of local tumors, SDT can trigger systemic immune responses by eliciting ICD to effectively inhibit distant metastatic tumors and prevent tumor recurrence. ICD has been reported to promote the processes of antigen uptake, processing, and presentation, thus facilitating the production of cytotoxic T cells (Shi and Lammers, 2019). Cai’s group constructed a multifunctional nanosonosensitizer by loading a manganese–protoporphyrin complex as a nanosonosensitizer into liposomes with surface modification of folic acid (FA) (Chen et al., 2021). FA is a targeting ligand that specifically binds to FA receptors overexpressed on the surface of many cancer cells (Li et al., 2013). Due to the decreased molecular orbital energy via metal coordination, the ^1^O_2_ generation efficacy of the formed FA-MnPs under US irradiation was greatly enhanced, which was also observed in the mimic tissue up to 8 cm depth. Such a good depth-responded action of FA-MnPs not only efficiently inhibited the growth of superficial tumors but also showed a good therapeutic effect for deep-seated tumors in 4T1 tumor-bearing mouse models. More importantly, FA-MnP–mediated SDT could repolarize macrophages from immunosuppressive M2 to antitumor M1 phenotypes. In addition, this treatment strategy triggered the activation of dendritic cells (DCs), T lymphocytes, and natural killer (NK) cells by eliciting ICD of tumor cells and reduced the population of regulatory T (Treg) cells. As such, a strong antitumor immunity was evoked in living mice, which resulted in the inhibition of tumor growth. Although this study provided a simple strategy for treatment of deep-seated and metastatic tumors, the tumors were not completely eradicated, and the therapeutic efficacy should be improved.

To induce robust ICD for cancer immunotherapy, Zhang and coworkers recently developed an SDT-based nanoplatform with continuous CO_2_ generation to enforce continuous US-induced inertial cavitation (UIC) for amplifying ROS production (Yin et al., 2021). The nanoplatforms were constructed by encapsulating CO_2_-adsorbed L-arginine (LA) and protoporphyrin (PpIX) as sonosensitizers into mesoporous organosilica nanoparticles (MONs). The formed nanoplatform (MON-PpIX-LA-CO_2_) could release CO_2_ in a continuous manner, thus forming the UIC effect under US irradiation, which enabled massive generation of ROS and afforded multiple enhancements of the SDT effect after a single administration. Such a high-efficiency ROS generation during MON-PpIX-LA-CO_2_–mediated treatment triggered robust ICD of tumor cells, thus promoting the maturation of DCs and activation of effector CD8^+^ T cells in 4T1 tumor-bearing mouse models. Meanwhile, the immunosuppressive M2 macrophages were polarized into antitumor M1 phenotypes. Due to the formed systemic antitumor immunity, the growth of both primary and distant metastatic 4T1 tumors was greatly inhibited.

Due to the dependence of oxygen for SDT, its therapeutic efficacy is often compromised by the hypoxic tumor microenvironment and continuous consumption of oxygen (Chen et al., 2017; Pan et al., 2020). By taking advantage of the SDT-aggravated hypoxia tumor microenvironment, Wang and Zhang’s group constructed a biomimetic decoy for SDT-induced enhanced ICD and immunotherapy (Zhao et al., 2020). The biomimetic decoy was constructed by encapsulating chlorin e6 (Ce6) as the sonosensitizers and a hypoxia-activated tirapazamine (TPZ) into pH-sensitive liposomes camouflaged by a red blood cell-platelet hybrid membrane. In the view of the immune escape and specific targeting behaviors of surface biomimetic membrane camouflages, the biomimetic decoy showed high accumulation and retention in tumor sites. Under US irradiation, Ce6 generated ROS for SDT and consumed oxygen to aggravate the tumor hypoxic microenvironment, which led to the activation of TPZ and thus achieved a highly effective synergistic therapy. As a result, such decoy-mediated therapy triggered amplified ICD of tumor cells and a strong antitumor immune response to inhibit the growth of B16-F10 tumors in C57BL/6 mice and prevent lung metastasis. Different from other therapeutic nanoagents, the biomimetic decoy retained platelet binding functions and thus could obviously inhibit the danger-associated molecular pattern (DAMP)–mediated tumor metastasis.

In addition to chemotherapy, ferroptosis has been combined with SDT to induce enhanced ICD and antitumor immunity. As an example, manganese porphyrin-based metal–organic frameworks (MOFs) containing metal ion zirconium (Zr) were constructed for SDT/ferroptosis/immunotherapy of hypoxic tumors (Xu et al., 2021). The MOFs showed a catalase-like activity to induce the decomposition of H_2_O_2_ in the tumor microenvironment to produce oxygen, thereby overcoming tumor hypoxia and enhancing the generation of ROS under US irradiation. In addition, Zr ions within MOFs decreased glutathione (GSH) levels in tumor cells to further promote ROS levels, thus achieving enhanced ROS-generated ferroptosis. Moreover, the MOF-mediated treatment under US irradiation greatly reversed the tumor immune microenvironment by increasing the populations of matured DCs and effector CD8^+^ T cells and decreasing the population of myeloid-derived suppressor cells (MDSCs) in tumor tissues. As such, the MOFs combined SDT, ferroptosis, and antitumor immunity, showing strong anticancer efficacy in both subcutaneous H22 tumors and metastatic 4T1 tumor models. However, the long-term biocompatibility of MOFs in living animals should be carefully investigated.

## Combinational Therapy of Sonodynamic Therapy With Immunotherapy

Although SDT can elicit antitumor immunity *via* the ICD effect, the strength is still limited for effective eradication of tumors (Li et al., 2021a). To achieve high therapeutic efficacy, SDT has been combined with different types of immunotherapy. For example, Liu’s group fabricated a two-dimensional (2D) coordination nanosheet composed of Zn^2+^ and tetrakis(4-carboxyphenyl) porphyrin (TCPP) with an excellent sonodynamic ROS generation for enhanced antitumor immunotherapy (Zhu et al., 2021). In addition, the 2D Zn-TCPP nanosheets exhibited a high surface area and thus could load cytosine phosphorothioate guanine oligodeoxynucleotide (CpG ODN), a potent toll-like receptor 9 (TLR9) agonist, as the immunotherapeutic agent. In the tumor tissue accumulated with Zn-TCPP/CpG, ROS was generated under US irradiation to induce ICD with the release of tumor debris as TAAs. CpG ODN agonists in nanosheets then worked together with the released TAAs to trigger a strong antitumor immune response. Therefore, Zn-TCPP/CpG nanosheet–mediated therapy could not only destroy primary tumors but also result in antitumor immunity to inhibit the growth of distant tumors in subcutaneous CT26 tumor–bearing mouse models. Such a combinational therapy also built a strong immunological memory effect to prevent tumor recurrence after treatment. Besides the good therapeutic efficacy, a key issue may be the possible leakage of CpG ODN agonists from nanosheets during blood circulation.

Different from TLR agonists, inhibitors of indoleamine 2,3-dioxygenase (IDO) can block important negative regulatory molecules overexpressed in tumor cells and antigen-presenting cells (Peng et al., 2018). Thus, the combination of SDT and IDO inhibitor–mediated immunotherapy has been adopted for cancer treatment. As demonstrated by Liu’s group, the authors developed a biomimetic nanosystem for the treatment of metastatic tumors via the synergetic action of SDT/CO gas therapy and IDO immunotherapy (Zhang D. et al., 2020). In their system, gold nanoparticles (AuNPs) were *in situ* synthesized on black phosphorus quantum dot (BPQD)–doped mesoporous silica frameworks (BMSNs) to form a hybrid nanoplatform (Au-BMSNs) acting as nanosonosensitizers, which were then loaded with CO-releasing molecules and the surface coated with a macrophage cell membrane. Such a biomimetic nanosystem showed an active targeting ability to tumors and thus achieved high tumor accumulation. Under ultrasonic irradiation, ^1^O_2_ was generated and CO was released, resulting in cell apoptosis and mitochondrial dysfunction of tumor cells. In addition, such an SDT/CO therapy could induce ICD of tumor cells and then trigger a potent antitumor immune response and long-term immune memory by combining with the IDO inhibitor-mediated immunotherapy. Such a combinational action effectively inhibited the growth of subcutaneous tumors, suppressed tumor recurrence, and also prevented lung metastasis in 4T1 tumor-bearing mouse models. However, the IDO inhibitors were intraperitoneally injected into mice, and their bioavailability should be further improved.

In addition to IDO inhibitors, the combination of SDT with immune checkpoint blockade using an anti-PD-1 or anti-PD-L1 antibody has emerged as another promising therapeutic strategy for cancer. Zhang and coworkers recently designed a transformable nanosensitizer with tumor microenvironment–activated SDT action and Ca^2+^ release for enhanced cancer immunotherapy (Tan et al., 2021). An acid-degradable CaP was coated onto the surface of titanium dioxide (TiO_2_) nanoparticles to form core/shell structured TiO_2_@CaP nanoplatforms as the nanosensitizers. In the acidic tumor microenvironment, the CaP shell was dissolved to release Ca^2+^, which led to mitochondrial dysfunction by overloading intracellular Ca^2+^. In addition, the disintegrated TiO_2_ nanoparticles allowed ROS generation under US irradiation for SDT. Such a cascade action enhanced the death of cancer cells and substantially amplified ICD, leading to enhanced activation and infiltration of tumor-specific cytotoxic T cells into 4T1 tumors of mice. After a combination with anti-PD-1–mediated checkpoint blockade therapy, TiO_2_@CaP nanoplatforms triggered a systemic antitumor immunity to inhibit the growth of both primary and distant tumors and suppress lung metastasis. This study indeed provided a smart tumor microenvironment–responsive nanosonosensitizers for effective cancer therapy.

In another study, Park’s group developed a necroptosis-inducible nanobubble (NB) to combine SDT and anti-PD-L1 immunotherapy for cancer (Um et al., 2020). Different from apoptosis, necroptosis is a caspase-independent necrosis with the characterizations of intracellular content leakages, plasma membrane integrity loss, and cytoplasmic swelling (Zhang et al., 2009). Necroptosis has been reported to boost antitumor immunity more efficiently than apoptosis (Suntharalingam et al., 2015). The NBs composed of perfluoropentane, carboxymethyl dextran, and Ce6 as the gas precursor, hydrophilic backbone, and hydrophobic sonosensitizer, respectively. Under US irradiation, NBs primarily induced ROS generation due to the action of Ce6 and then acoustic cavitation of perfluoropentane caused necroptosis by inducing burst-mediated cell membrane disintegration, leading to ICD of tumor cells. Such an action further triggered antitumor immunity by promoting the maturation of DCs and activation of CD8^+^ cytotoxic T cells. By combining with immune checkpoint blockade (anti-PD-L1), the NB-mediated therapy achieved complete regression of the primary tumors and effective inhibition of metastatic tumors in CT26 tumor–bearing mouse models.

To overcome the limitations of SDT, the combination of SDT and chemodynamic therapy (CDT) with immune checkpoint therapy can be adopted. As an example, a multifunctional nanoreactor of polyethylene glycol (PEG)–modified CoFe_2_O_4_ nanoflowers (CFPs) was synthesized using a typical solvothermal method for an augmented SDT/CDT-elicited robust immune response (Fu et al., 2021). The CFP showed a catalase-like activity to produce a hydroxyl radical (·OH) for CDT and catalyze the decomposition of H_2_O_2_ in a tumor microenvironment to form O_2_ to relieve tumor hypoxia, which enabled the generation of a high level of ^1^O_2_ under US irradiation to achieve effective SDT. Thus, CFP-mediated SDT/CDT could efficiently induce ICD of tumor cells, which would lead to a boost in antitumor immunity in 4T1 tumor–bearing mice after the combination with immune checkpoint blockade (anti-PD-L1). Such a combinational therapy afforded an excellent efficacy in inhibiting the growth of primary and distant tumors and suppressing lung metastasis. Different from the aforementioned nanosystems, CFP could be used for T_2_-weighted magnetic resonance (MR) imaging of tumors and thus achieved the evaluation of tumor accumulation for US irradiation.

In another study, a nanosystem based on L-arginine (LA)–loaded black mesoporous titania (BMT) was fabricated for SDT and gas therapy–combined immunotherapy (Wang et al., 2021). In this system, BMT and LA acted as a sonosensitizer and exogenous NO supplementation to produce ^1^O_2_ for SDT and NO for gas therapy under US irradiation, respectively. Moreover, more NO was produced because of the oxidation of LA promoted by the generated ^1^O_2_. The high levels of ^1^O_2_ and NO led to strong intracellular oxidative stress and DNA double-strand breaks, which induced apoptosis of cancer cells with the release of TAAs. By combining with the immune checkpoint inhibitor (anti-PD-L1), a strong antitumor immune response was induced to effectively treat both primary and distant tumors and inhibit lung metastasis of U14 tumors.

**FIGURE 1:**
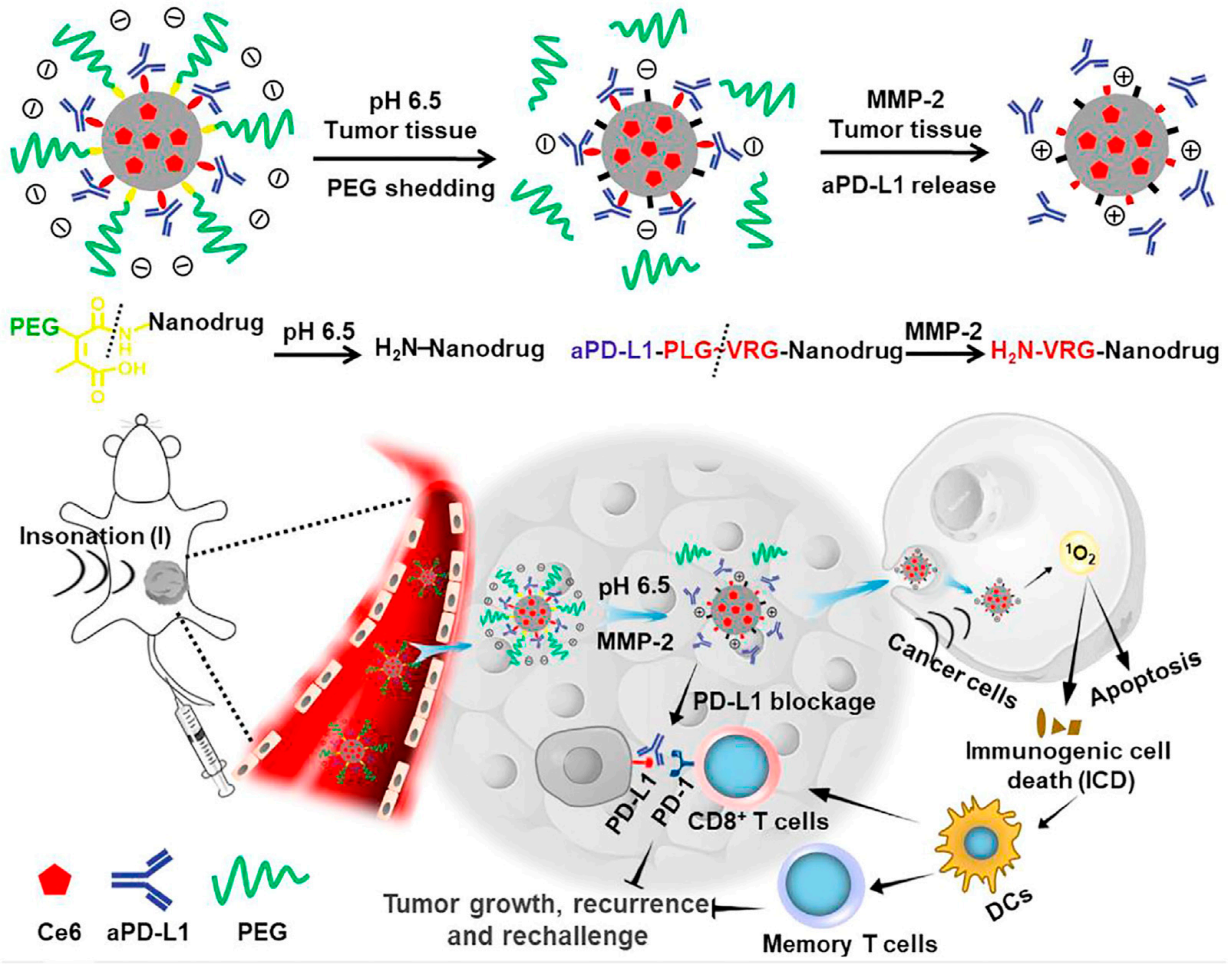
Schematic illustration of the sensitivity and *in vivo* performance of the pH and MMP-2 dual-sensitive PEG-coated nanodrugs for tumor-targeting combinational SDT and immunotherapy of cancer. Reproduced, with permission, from Huang et al. (2021). Copyright 2021, Elsevier.

Although the combination of SDT and immunotherapy has achieved ideal antitumor efficacy, the administration of immunotherapeutic agents (such as anti-PD-1 and anti-PD-L1) is conducted via systematic injection. This often leads to insufficient accumulation of immunotherapeutic agents in tumor sites, which greatly inhibits the antitumor efficacy and potentially causes immune-related adverse effects (Wang et al., 2018). To address this concern, Shuai’s group developed a tumor microenvironment–responsive nanodrug for combinational SDT/immunotherapy (Huang et al., 2021). Nanodrugs were constructed based on lipid nanocarriers with the encapsulation of Ce6 in the core as the sonosensitizers, a conjugation of an anti-PD-L1 antibody via MMP-2–cleavable peptides, and PEG chains through an acid-labile amide bond (Figure 1). In acidic tumor tissues, the PEG outer chains were shed from nanodrugs to promote the cellular uptake by tumor cells, and the overexpressed intratumoral MMP-2 cleaved the peptide linkers to trigger the release of anti-PD-L1. In addition, Ce6 within nanodrugs mediated SDT under US irradiation to induce ICD. As a result, the nanodrug-enabled combinational SDT/immunotherapy elicited a robust antitumor immunity and long-term immune memory, resulting in effective inhibition of tumor growth and suppression of tumor recurrence in B16-F10 tumor–bearing mice. Moreover, this therapeutic strategy remarkably reduced systemic immune–related adverse effects and thus showed great promise for cancer therapy.

## Combinational Therapy of Sonodynamic Therapy With Multiple Immunotherapies

To obtain better therapeutic efficacies by triggering stronger antitumor immunity, some nanosystems integrating two types of immunotherapeutic agents have been developed for SDT-combined multiple immunotherapies. Wang and coworkers constructed a multifunctional nanosonosensitizer by loading Ce6 and CpG ODN in TiO_2_ nanoparticles. Due to the existence of TiO_2_-Ce6 for augmented SDT under US irradiation and immune adjuvant CpG to boost the immune response, the nanosonosensitizers (TiO_2_-Ce6-CpG) effectively ablated tumors with the release of TAAs and stimulated the immune system to activate the adaptive immune responses. After combining with anti-PD-L1, the TiO_2_-Ce6-CpG–mediated therapy induced a strong antitumor immune response to greatly inhibit the growth of treated primary tumors and non-treated distant tumors. Actually, the antitumor efficacy still should be further improved as all the treated mice died after around 80 days.

To achieve the targeting delivery of nanosystems into tumor sites, Yang and coworkers designed a cell membrane–coated biomimetic nanovaccine to combine SDT with multiple immunotherapies (Zhan et al., 2021). The nanovaccine was constructed by integrating manganese porphyrin–based MOF (Mn-MOF) with CpG ODN, followed by surface coating with cell membranes derived from ovalbumin (OVA)-overexpressing melanoma B16 cells. The nanovaccine showed a prolonged blood circulation and enhanced tumor targeting ability to hypoxic tumors, in which the high level of ROS was generated under US irradiation for SDT accompanied by ICD of tumor cells. SDT-induced TAAs and OVA on a cell membrane showed a vaccine-like function and synergized with CpG ODN to trigger a strong tumor-specific immune response by promoting the maturation of DCs and activation of T cells. After combining with the anti-PD-1–mediated immunotherapy, the nanovaccine-triggered therapeutic action induced a stronger systemic immune response and long-term immunological memory function to inhibit the growth of primary and distant tumors and prevent recurrence in subcutaneous B16-OVA tumor–bearing mice. This study indeed provided a promising strategy for the combinational therapy with immune checkpoint inhibitors in hypoxic tumors.

**FIGURE 2:**
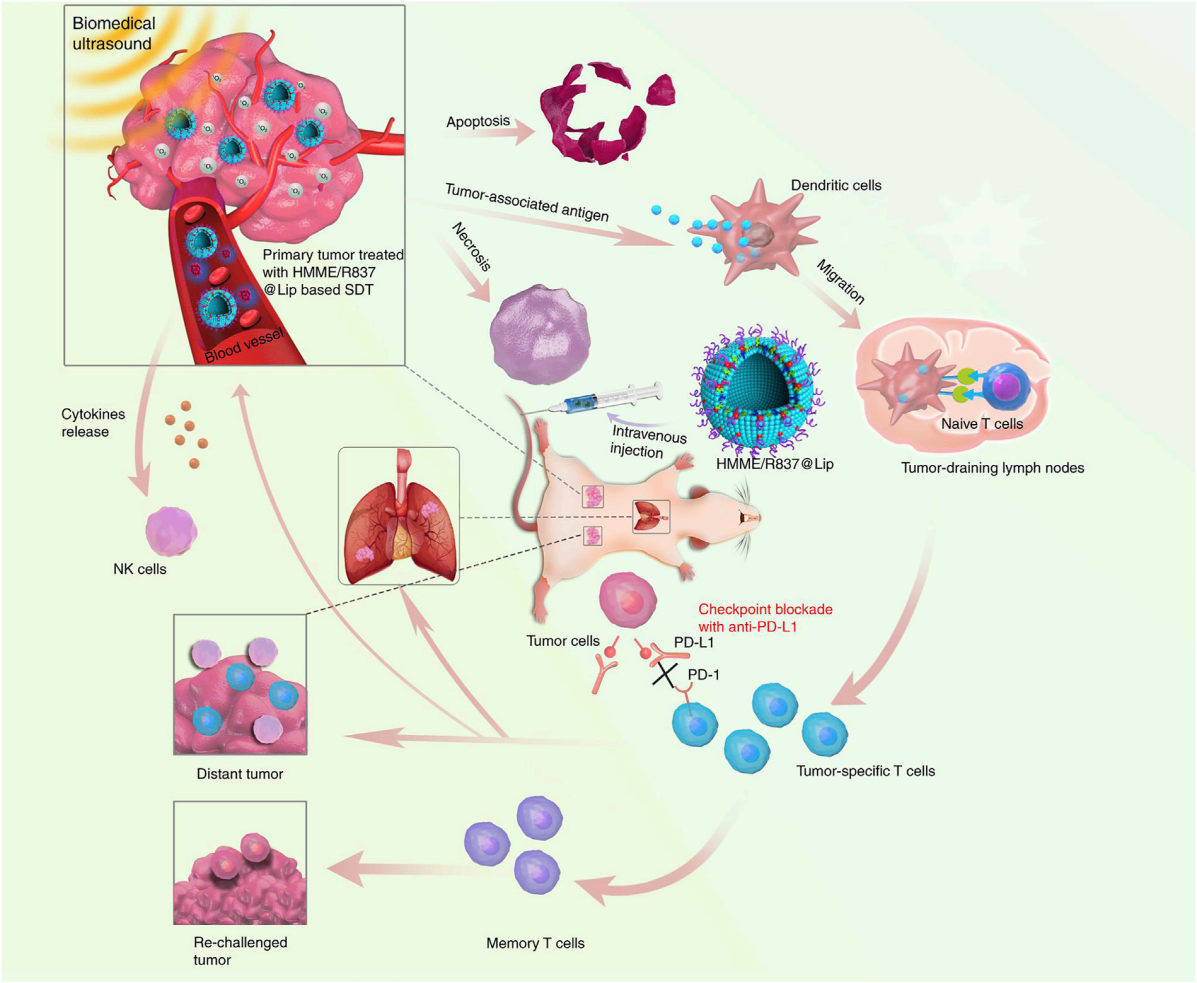
Design principle of nanosonosensitizer-augmented synergistic SDT and immunotherapy. Schematic illustration of antitumor immune responses induced by combined noninvasive SDT with immune adjuvant–contained nanosonosensitizer and checkpoint blockade for effective cancer immunotherapy. Reproduced, with permission, from Yue et al. (2019). Copyright 2019, Nature Publishing Group.

By using a TLR7 agonist, imiquimod (R837), as the immune adjuvant, Chen’s group reported nanosonosensitizers to allow SDT, enhanced SDT-based immunotherapy, and anti-PD-L1 for cancer treatment (Yue et al., 2019). The nanosonosensitizers were constructed by loading hematoporphyrin monomethyl ether (HMME) as the sonosensitizer and immune adjuvant (R837) into nano-liposomes. After accumulation in tumor sites, the nanosonosensitizers generated ^1^O_2_ under US irradiation to induce apoptosis/necrosis and ICD of tumor cells with the release of TAAs (Figure 2). The *in situ* released TAAs showed a vaccine-like function together with R837, leading to the elicitation of antitumor immunity by promoting maturation of DCs, activation of T cells, and secretion of cytokines. The strength of immune response was greatly enhanced after combining with anti-PD-L1–mediated immune checkpoint blockade as the population of tumor-infiltrating CD8^+^ T cells increased. Such a combinational therapy could treat primary tumors and prevent the progression of tumor metastasis in subcutaneous CT26 colorectal cancer and orthotopic 4T1 breast cancer mouse models. In addition, the therapeutic action provided a long-term immunological memory function to protect against tumor recurrence. More importantly, all the major components in nanosonosensitizers are clinically approved for use; this combinational therapeutic strategy is thus highly promising for further clinical translation.

## Discussion

Recent development of nanosonosensitizers with US-induced ROS generation to combine SDT with immunotherapy for cancer treatment has been summarized. Although the efficacies of SDT are often limited by a hypoxic tumor microenvironment, different strategies have been adopted to address this issue, such as the production of molecular oxygen (Zhang Y. et al., 2020), supplement of oxygen (Zeng et al., 2020), and depletion of GSH (Dong et al., 2020). In addition to direct ablation of tumors, SDT can induce ICD of tumor cells, thus promoting the activation of immune responses. Further combination of SDT with different types of immunotherapy will lead to obviously enhanced antitumor immunity. As a result, this combinational therapy can afford enhanced therapeutic efficacy in suppressing tumor growth and metastasis, and even preventing tumor recurrence.

Although these are promising results, several concerns of SDT-combined immunotherapy using nanosonosensitizers still need to be considered before the successful translation for clinical trials. First, US has a stronger tissue penetration ability relative to light (Qian et al., 2016), while SDT-combined immunotherapy has not been explored to treat deep-seated tumors in living mice, which should be systematically evaluated in the future. Second, most of immunotherapeutic agents are systemically injected into mice and often have limited accumulation in tumor sites, leading to low therapeutic efficacy and potential immune-related adverse effects. Loading immunotherapeutic agents in nanocarriers to achieve their targeting delivery into tumors can commendably address these issues (Uthaman et al., 2020; Li et al., 2021b). Third, the metabolism and degradation of nanoparticles in living animals should be carefully evaluated to ensure their long-term biosafety. It is of high demand to develop biodegradable or clearable nanoparticles for the therapeutic purposes (Li and Pu, 2019). Fourth, the dynamic monitoring of immune responses in living animals after treatments is necessary to ensure effective therapy, which however is a great challenge. The integration of immune-specific imaging reporters into nanosystems is a feasible method to achieve monitoring of immune activation (He et al., 2020; Ramesh et al., 2020). Fifth, some efforts should be put to combine SDT with cancer vaccines or adoptive T-cell therapy. In addition, the potential applications of SDT-combined immunotherapy using nanosonosensitizers for the treatment of other diseases such as infectious diseases and autoimmunity can be explored (Pang et al., 2019).
